# Transient Two-Layer Electroosmotic Flow and Heat Transfer of Power-Law Nanofluids in a Microchannel

**DOI:** 10.3390/mi13030405

**Published:** 2022-03-01

**Authors:** Shuyan Deng, Tan Xiao

**Affiliations:** Institute of Architecture and Civil Engineering, Guangdong University of Petrochemical Technology, Maoming 525011, China; xiaotan@gdupt.edu.cn

**Keywords:** transient two-layer flow, electroosmotic flow, power-law nanofluid, heat transfer, Laplace transform, nanoparticle volume fraction

## Abstract

To achieve the optimum use and efficient thermal management of two-layer electroosmosis pumping systems in microdevices, this paper studies the transient hydrodynamical features in two-layer electroosmotic flow of power-law nanofluids in a slit microchannel and the corresponding heat transfer characteristics in the presence of viscous dissipation. The governing equations are established based on the Cauchy momentum equation, continuity equation, energy equation, and power-law nanofluid model, which are analytically solved in the limiting case of two-layer Newtonian fluid flow by means of Laplace transform and numerically solved for two-layer power-law nanofluid fluid flow. The transient mechanism of adopting conducting power-law nanofluid as a pumping force and that of pumping nonconducting power-law nanofluid are both discussed by presenting the two-layer velocity, flow rates, temperature, and Nusselt number at different power-law rheology, nanoparticle volume fraction, electrokinetic width and Brinkman number. The results demonstrate that shear thinning conducting nanofluid represents a promising tool to drive nonconducting samples, especially samples with shear thickening features. The increase in nanoparticle volume fraction promotes heat transfer performance, and the shear thickening feature of conducting nanofluid tends to suppress the effects of viscous dissipation and electrokinetic width on heat transfer.

## 1. Introduction

It is well known that in microchannels the contact between the electrolyte solution and the solid surface of the channel wall leads to the rearrangement of charged ions, inducing an electric double layer (EDL) near the channel wall. In the presence of EDL, a layer of conducting fluid under a tangentially-applied electric field moves forward, forming electroosmotic flow (EOF); this phenomenon is called electroosmosis. Due to such favorable attributes as its ease of integration, plug-like profile, and the independence of its non-mechanical parts, the electroosmosis pumping mechanism has become a common transport phenomena in microfluidic devices [1]. In order to meet the growing demand for electroosmosis-based applications, a large number of works have theoretically studied EOF from different point of view. The transport characteristics of EOF in containers with different geometries, including slit microchannels [2], microtubes [3,4], rectangular microchannels [5], elliptic microchannels [6], and T-shaped microchannels [7] have been investigated. Several working liquids in microdevices, such as biomedical lab-on-a-chip, show nonlinear rheological behaviors, which is where the use of non-Newtonian fluid modeling becomes relevant. The nonlinear relationship between the shear stress and shear rate has been carefully treated using the power-law model [8,9], Casson model [10], Maxwell model [11], Carreau model [12], etc. The power-law model was first proposed by Das and Chakraborty [8] to describe the rheological behavior of blood, which has received great attention due to its wide coverage and the simple rheological relation [2,13]. The various aspects of power-law fluid for EOF have been discussed; the power-law model incorporates the shear thinning rheological behavior encountered in DNA solutions, and shear thickening rheological behavior encountered in cornstarch solution [14]. Recently, external environmental effects on EOF, such as a rotating frame or peristalsis, have been considered. In microchannel flow, a rotating environment induces Coriolis force, which causes a secondary flow; this is applied in biofluid transportation, drug delivery, and DNA analysis [15]. The theoretical analysis of rotating EOF was first studied by Chang and Wang [16], and has subsequently been extended in literature [17,18]. In order to obtain a comprehensive understanding of the intricate mechanism of rotational flow for biofluid flow, Kaushik et al., engaged in a transient analysis of the rotational electrohydrodynamics of power-law fluids under the effect of EDL [19]. Moreover, in the application of EOF to biomedical and biochemical analysis, peristalsis is introduced to assist in the EOF of biofluids, and thus electroosmosis-modulated peristaltic flow has recently become a frequently-studied research topic [20].

Owing to the application of external electric fields in electroosmosis-driven flow systems, the applied electric voltage leads to an inherent byproduct of the Ohmic resistance of electrolytes, namely, the Joule heating effect. Joule heating-induced heat transfer exerts an influence on transport performance by altering the electric properties of working liquids, especially for certain thermally-liable samples, which has been widely discussed in numerous works [21,22,23,24,25]. To optimize the hydrodynamic transport process and minimize the Joule heating effect, combined electric and magnetic fields are applied to working liquids in order to improve the actuation mechanism in microfluidics, which has the advantage of lower voltage operation, convenient manufacture, and the independence of moving parts [26,27]. On the other, to promote heat transfer and reduce entropy generation in the heat exchange equipment of microfluidics, nanofluid is created by adding nanosized metal particles, which possesses boosted thermal conductivity compared to conventional pure fluids, to the sample [28,29,30]. Nanofluid flow has been extensively applied in different fields, as it has none of the usual drawbacks such as sedimentation, blockage, and pressure drop [31,32,33]. AI_2_O_3_–water Nanofluid is used for cooling microprocessors or other microelectronic components due to its enhanced thermal conductivity [34], which exhibits shear thinning rheological behavior in certain ranges of nanoparticle volume fraction [35]. Moreover, in order to provide a better understanding of blood flow and other non-Newtonian biological flows in biomicrofluidic chips (such as pseudoplastic aqueous nanoliquid flow driven by electroosmosis and peristalsis and Cu/CuO–blood microvascular nanoliquid flow under thermal, microrotation, and electromagnetic field effects) were studied in [36,37], respectively. Carboxymethyl cellulose (CMC) water with γ-AI_2_O_3_, TiO_2_ and CuO particles has been experimentally investigated for the achievement of efficient thermal management in microelectronics [38]. A comprehensive literature survey of topics in nanofluid flow indicates that the viscosity of several nanofluids mentioned above shows a nonlinear dependence on the shear rate and volume fraction of nanoparticles; thus, the power-law nanofluid model is proposed to precisely describe the rheological behavior of such nanofluids [39,40,41,42]. Regarding the power-law nanofluid, the heat transfer characteristics in magneto-hydrodynamic flow [43], convection flow [44], and EOF [45,46,47] have been extensively investigated.

The great advent of technologies in microelectrical mechanical systems (MEMS) enables the delivery, mixing, or separation of multi-liquids at microscales. However, certain liquids, for instance oil, blood, and ethanol, have low electrical conductivity (<10^−^^6^ S m^−^^1^) and are defined as nonconducting fluids [48], which fail to be driven by electroosmosis. Furthermore, for certain liquids the applied electric voltage leads to undesirable problems such as the generation of gases, fluctuation of PH value, or electrochemical decomposition. In this context, Brask et al. developed a two-layer flow system where the conducting fluid driven by electroosmotic force is adopted as driving mechanism to drag the nonconducting fluid; this has gained great attention in recent decades [49]. The steady hydrodynamic behaviors of two-Newtonian fluid EOF in a rectangular microchannel [50], two-power-law fluid combined electroosmotics with pressure driven flow in a microtube [51], and Newtonian–Casson fluid EOF in a microtube [48] have all been theoretically studied in this context. In terms of transient hydrodynamical behaviors, Gao et al. characterized the transient two-layer EOF of Newtonian fluids in a rectangular microchannel by presenting analytical velocities and flow rates at different viscosities and different electroosmotic properties [52]. Su et al., presented semi-analytical velocities for two-layer combined electroosmotic and pressure driven flow of Newtonian fluids in a slit microchannel at different electric and hydrodynamic parameters [53]. Time periodic transport characteristics of two-Newtonian liquid combining electroosmotic and pressure driven flows in a microtube have been studied numerically [54]. In order to improve transport efficiency and reduce the Joule heating effect in a two-layer pumping system, a magnetic field can be applied in addition to the pressure gradient to form a magneto-hydrodynamic EOF; the corresponding entropy generation analysis has been conducted as well [55,56]. Two-layer EOF assisted by peristalsis force was proposed by Ranjit et al., who analyzed entropy generation and heat transfer in such cases [57]. External factors such as a rotating environment [58] or varying wall shapes together with zeta potential [59] have been considered in two-layer electroosmotic systems, and the resulting influence on hydrodynamic behavior has been discussed. Furthermore, because of its desirable thermal conductivity properties, nanofluids can be applied in two-layer mixed convection flows, which are characterized by the power-law nanofluid model; the outcomes can help with the promotion of heat transfer performance [60]. Entropy generation and heat transfer in immiscible EOF of two conducting power-law nanofluid flows through a microtube have been analyzed by computing their temperature, Nusselt number, and entropy generation at different nanoparticle volume fractions and different rheological and electroosmotic properties [61]; the rheological effect of the peripheral fluid plays a dominant role in thermal performance as compared to that of inner fluid.

The application of chemical mixing/separation in thermofluidic micropumps has become increasingly frequent; therefore, the corresponding microscale cooling and heat exchangers need to be carefully designed as working liquids combined with nanoparticles show nonlinear rheological behavior in nature. To the authors’ best knowledge, the underlying mechanism of the transient transport process as it develops from an unsteady to a steady state in two-power-law nanofluid EOF in a slit microchannel remains to be discovered. Therefore, this paper studies transient two-layer flow with one layer of conducting power-law nanofluid and one layer of nonconducting power-law nanofluid in a slit microchannel, with consideration of Joule heating and viscous dissipation. The governing equations are established based on the Cauchy momentum equation, continuity equation, energy equation, and power-law nanofluid rheological relation, which are analytically solved for two-Newtonian fluid flow and numerically solved for two-power-law nanofluid flow. For the hydrodynamic aspect, the mechanisms involved in using power-law nanofluid as a pumping force as well as those of pumping power-law nanofluid as the system develops from unsteady to a steady state are carefully discussed by presenting the time evolution of velocity and flow rate at different parameters. For the thermal aspect, in order to guarantee efficient thermal performance, the heat transfer characteristics arising from the interplay of the nanoparticles, power-law rheological behavior, viscous dissipation, and electrokinetic effects in a two-layer system are analyzed theoretically. The results are relevant for assisting in determining the operating parameters for optimal performance of microdevices characterized by multi-fluid delivery, mixing, or cooling.

## 2. Problem Formulation

### 2.1. Electric Potential Distribution

**Figure 1:**
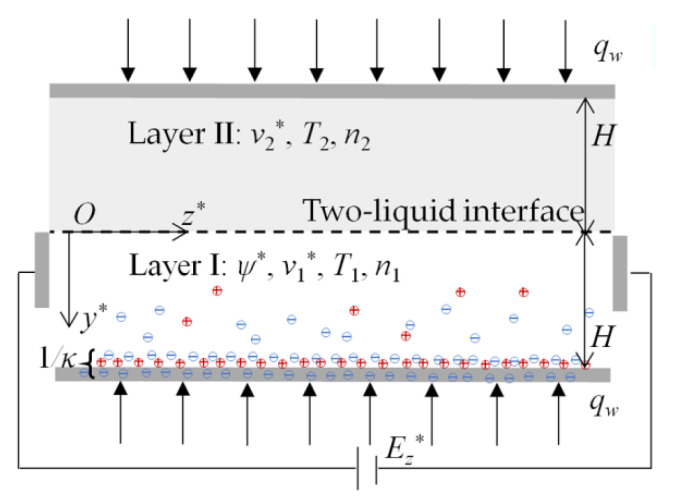
Schematic of two-layer EOF of power-law nanofluids in a slit microchannel.

A two-layer immiscible EOF of power-law nanofluids in a slit microchannel is considered where the power-law nanofluid in layer I is conducting and the power-law nanofluid in layer II is nonconducting (Figure 1). The AI_2_O_3_ nanoparticles are suspended and uniformly distributed in a two-layer power-law base fluid system where carboxymethyl cellulose–water (CMC-water) can be represented by shear thinning fluid [38] and, without loss of generality, the base fluids fall into the categories of a shear thinning fluid and a shear thickening one. According to previously published work [50], the zeta potential difference near the two-liquid interface (y^*^ = 0) is negligible. The heights of layer I and layer II are represented by *H*. In the two-layer system, the EDL forms near the channel wall within the region of layer I, which creates the electric potential distribution ψ^*^. When a uniform electric field $E_{z}^{*}$ is tangentially exerted across layer I, the conducting power-law nanofluid moves forward under the electroosmotic force due to the existence of EDL near the lower channel wall, which drags the nonconducting power-law nanofluid in layer II via the interfacial viscous stress, eventually forming a two-layer EOF.

It is assumed that the zeta potential ζ^*^ is small and the EDL thickness is far less than the height of microchannel; thus, the EDLs near the channel walls will not overlap. Eventually, based on electrostatic theory, the electric potential distribution ψ^*^ is governed by the well-known Poisson–Boltzmann (P-B) equations [50]

(1) $$\frac{d^{2}\psi^{*}}{dy^{*2}}=-\frac{\rho_{e}}{\varepsilon }$$

(2) $$\rho_{e}=-2n_{0}z_{0}e\sinh\left(\frac{z_{0}e\psi^{*}}{k_{B}T_{0}}\right)$$

According to the established model in the scientific literature [32,62], when nanoparticles with an order of nm and with a volume fraction of *ϕ* ≤ 10% are distributed in the channel with μm-sized height, it is physically reasonable to assume that the EDLs around the nanoparticles are rather small compared to the EDLs near the channel walls, and can thus be neglected, as well as that there is no electrophoretic force on the nanoparticles. Correspondingly, the nanoparticles have no influence on the local electric charge density *ρ_e_*, which can be governed by the P-B equations, and their influence on liquid property can be incorporated into the effective viscosity and effective thermal conductivity of the nanofluid [28,29,30] such that their importance can be attached to the effect of power-law feature and the effect of nanoparticles on the hydrodynamic and thermal characteristics of transient two-layer flow.

The P-B equations are subject to the following boundary conditions:

(3) $${\frac{d\psi^{*}}{dy^{*}}|}_{y^{*}=0}=0,\;{\psi^{*}|}_{y^{*}=H}=\zeta^{*}$$

where *ε* denotes the dielectric constant, *n*_0_ denotes the ion density, *z*_0_ denotes the valence, *e* is the electron charge, and *k_B_* and *T*_0_ represent the Boltzmann constant and the absolute temperature, respectively.

Introducing the nondimensional variables *ψ = ez*_0_ψ^*^/(*k_B_T*_0_), *ζ* = *ez*_0_ζ^*^/(*k_B_T*_0_), and *y* = y^*^/*H*, *K* = *κH* with *κ*^2^ = 2*e*^2^z_0_^2^*n*_0_/(*εk_B_T*_0_), and applying Debye–Hückel approximation (sinh*ψ* ≈ *ψ* [50]) to Equations (1)–(3) when |ζ^*^| ≤ 0.025 V, the nondimensional versions of the P-B equations can be rewritten as

(4) $$\frac{d^{2}\psi }{dy^{2}}=K^{2}\psi$$

(5) $${\frac{d\psi }{dy}|}_{y=0}=0,\;{\psi |}_{y=1}=\zeta$$

Solving Equations (4) and (5) yields the electric potential distribution, as below:

(6) $$\psi =\frac{\zeta \cosh(Ky)}{\cosh(K)}$$

With Equation (6), the electroosmotic force driving the conducting nanofluid can be obtained.

### 2.2. Two-Layer Velocity Distribution and Flow Rates

Focusing on the hydrodynamical aspects of transient two-layer EOF of power-law nanofluids in a slit microchannel, the governing equations for velocity distribution are represented by the Cauchy momentum equation and the continuity equation, as below:

(7) $$\nabla \cdot {\vec{v}}^{*}=0$$

(8) $$\rho \left[\frac{\partial {\vec{v}}^{*}}{\partial t^{*}}+\left({\vec{v}}^{*}\cdot \nabla \right){\vec{v}}^{*}\right]=\nabla \cdot \vec{\tau }+\vec{f}-\nabla p$$

where $\vec{v}$ is the velocity vector, *ρ* is the liquid density, t^*^ is the time, τ is the shear stress, $\vec{f}$ denotes the body force vector, and ∇p denotes the pressure gradient. The channel is open-ended and no pressure gradient is induced. The following assumptions are made for the purpose of analysis: (1) the properties of the nanofluids are independent of the external electric field, ion concentration, and temperature [48,50]; (2) the two-layer flow is immiscible, laminar, and incompressible, and the two-liquid interface remains distinguishable [48,50]; and (3) the gravity force and buoyancy force of the nanofluids are neglected [32]. As a result, there is only velocity component along z^*^ direction v^*^_i_ (y^*^,t^*^), with *i* = 1,2 and the body force equal to the electroosmotic force, *f_z_* = *E_z_ρ_e_*. In a system with two power-law nanofluids flowing through a slit microchannel, the shear stress of a power-law nanofluid is *τ_i_* = *η_effi∙_*∂v^*^_i_/∂y^*^, where *η_effi_* implies the effective dynamic viscosity of the power-law nanofluid, which nonlinearly depends on the shear rate ∂v^*^_i_/∂y^*^ and the nanoparticle volume fraction *ϕ*, namely, $\eta_{effi}=m_{0}^{*}/{\left(1+\phi \right)}^{5/2}\cdot |\partial v_{i}^{*}/\partial y^{*}{|}^{n_{i}-1}$ [13,28,43,51], where *m*_0_^*^ is the consistency viscosity coefficient, *n_i_* is the flow behavior index, the subscript *i* = 1 represents the conducting nanofluid in layer I, and *i* = 2 represents the nonconducting nanofluid in layer II. Note that *n_i_* < 1 corresponds to shear thinning base fluid, *n_i_* = 1 corresponds to Newtonian base fluid and *n_i_* > 1 corresponds to shear thickening base fluid. Accordingly, the modified Cauchy momentum equation for the conducting power-law nanofluid in layer I under the electroosmotic force is expressed as

(9) $$\frac{\partial v_{1}^{*}}{\partial t^{*}}=\frac{m_{0}^{*}}{{\left(1+\phi \right)}^{5/2}\rho_{1}}\frac{\partial }{\partial y^{*}}\left({\left|\frac{\partial v_{1}^{*}}{\partial y^{*}}\right|}^{n_{1}-1}\frac{\partial v_{1}^{*}}{\partial y^{*}}\right)+\frac{1}{\rho_{1}}\rho_{e}E_{z}^{*}\;\mathrm{for}\;0\leq y^{*}\leq H$$

The modified Cauchy momentum equation of nonconducting power-law nanofluid in layer II is expressed as

(10) $$\frac{\partial v_{2}^{*}}{\partial t^{*}}=\frac{m_{0}^{*}}{{\left(1+\phi \right)}^{5/2}\rho_{2}}\frac{\partial }{\partial y^{*}}\left({\left|\frac{\partial v_{2}^{*}}{\partial y^{*}}\right|}^{n_{2}-1}\frac{\partial v_{2}^{*}}{\partial y^{*}}\right)\;\mathrm{for}-H\leq y^{*}\leq 0$$

The velocities and shear stresses of the two-layer liquid satisfy the matching conditions, the velocities at the channel walls satisfy the no-slip condition, and the two-layer flow is initially set as motionless [55], thusly:

(11) $$v_{1}^{*}{|}_{y^{*}=0}=v_{2}^{*}{|}_{y^{*}=0},\;\eta_{eff1}\cdot {\frac{\partial v_{1}^{*}}{\partial y^{*}}|}_{y^{*}=0}=\eta_{eff2}\cdot {\frac{\partial v_{2}^{*}}{\partial y^{*}}|}_{y^{*}=0},\;v_{1}^{*}{|}_{y^{*}=H}=0,\;v_{1}^{*}{|}_{y^{*}=-H}=0,\;v_{i}^{*}{|}_{t^{*}=0}=0$$

With the introduction of the nondimensional variables *t* = t^*^m_0_/(*ρ*_1_*H*^2^), *v_i_* = v^*^_i_/*U*, and *y* = y^*^/*H*, by replacing Equations (2) and (6) with Equations (9)–(11), the nondimensional versions of the governing equations can be obtained as follow

(12) $$\frac{\partial v_{1}}{\partial t}=\frac{m_{1}}{m_{0}}\frac{\partial }{\partial y}\left({\left|\frac{\partial v_{1}}{\partial y}\right|}^{n_{1}-1}\frac{\partial v_{1}}{\partial y}\right)-\frac{GE_{z}\zeta }{\cosh(K)}\cosh(Ky)\;\mathrm{for}\;0\leq y\leq 1$$

(13) $$\frac{\partial v_{2}}{\partial t}=\rho_{r}\frac{m_{2}}{m_{0}}\frac{\partial }{\partial y}\left({\left|\frac{\partial v_{2}}{\partial y}\right|}^{n_{2}-1}\frac{\partial v_{2}}{\partial y}\right)\;\mathrm{for}-1\leq y\leq 0$$

(14) $$v_{1}{|}_{y^{}=1}=v_{2}^{}{|}_{y^{}=-1}=0,v_{1}^{}{|}_{y^{}=0}=v_{2}^{}{|}_{y^{}=0},m_{1}{\left|\frac{\partial v_{1}^{}}{\partial y^{}}\right|}^{n_{1}-1}\cdot {\frac{\partial v_{1}^{}}{\partial y^{}}|}_{y^{}=0}=m_{2}{\left|\frac{\partial v_{2}^{}}{\partial y^{}}\right|}^{n_{2}-1}\cdot {\frac{\partial v_{2}^{}}{\partial y^{}}|}_{y^{}=0},v_{i}{|}_{t=0}=0$$

where $m_{i}={\left(U/H\right)}^{n_{i}-1}m_{0}/{\left(1-\phi \right)}^{5/2}$, *ρ_r_* = *ρ*_2_/*ρ*_1_, *G* = 2*zen*_0_ζ^*^/(*ρ*_1_*U*^2^), and *E_z_* = E^*^_z_HRe/ζ^*^, with *Re* = ζ^*^UH/*m*_0_, *U* the reference velocity.

With the nondimensional transient velocities *v*_1_ and *v*_2_ solved as in Equations (12)–(14), the transient flow rates are defined as follows:

(15) $$Q_{1}=\int_{0}^{1}v_{1}dy,\;Q_{2}=\int_{-1}^{0}v_{2}dy$$

As time elapses, the transient velocities for layer I and layer II, namely, *v*_1_ and *v*_2_, reach steady status, and are then expressed as $v_{s1}(y)=\underset{t\to \infty }{\lim}v_{1}(y,t)$ and $v_{s2}(y)=\underset{t\to \infty }{\lim}v_{2}(y,t)$. To compare the flow rate of the conducting nanofluid in layer I (flow rate I) and the nonconducting nanofluid in layer II (flow rate II) with different parameters, the steady flow rate ratio is defined as

(16) $$Q_{r}=\frac{\int_{0}^{1}v_{s1}dy}{\int_{-1}^{0}v_{s2}dy}$$

### 2.3. Two-Layer Temperature Distribution and Heat Transfer Rate

With the steady velocity distribution obtained from Equations (12)–(14), the temperature distribution for the thermally fully developed two-layer flow can be determined from the following energy equation:

(17) $${\left(\rho c_{p}\right)}_{eff}\left(\frac{\partial T}{\partial t^{*}}+{\vec{v}}_{s}^{*}\cdot \nabla T\right)=k_{eff}\nabla^{2}T_{}+\lambda E_{z}^{*}{}^{2}+\eta_{eff}\Phi^{*}$$

where *T* denotes the temperature distribution, ${\vec{v}}_{s}$ means the steady velocity vector, *c_p_* means the specific heat at constant pressure, *k* is the thermal conductivity, *λ* is the electric conductivity of base fluid, Φ^*^ measures the viscous dissipation effect, and the subscript *eff* means the nanofluid.

The assumption that the two-layer flow is fully thermally developed leads to the vanishing of the unsteady part of Equation (17), *∂T*/∂t^*^, hence producing the following energy equations for the conducting nanofluid and nonconducting nanofluid, respectively, along with their corresponding boundary conditions [26,55]:

(18) $${\left(\rho c_{p}\right)}_{eff}{}_{1}v_{s1}^{*}\frac{\partial T_{1}}{dz^{*}}=k_{eff}{}_{1}\frac{d^{2}T_{1}}{dy^{*}{}^{2}}+\lambda E_{z}^{2}+\eta_{eff}{}_{1}{\left(\frac{dv_{s1}^{*}}{dy^{*}}\right)}^{2}\;\mathrm{for}\;0\leq y^{*}\leq H$$

(19) $${\left(\rho c_{p}\right)}_{eff}{}_{2}v_{s2}^{*}\frac{\partial T_{2}}{\partial z^{*}}=k_{eff}{}_{2}\frac{d^{2}T_{2}}{dy^{*}{}^{2}}+\eta_{eff}{}_{2}{\left(\frac{dv_{s2}^{*}}{dy^{*}}\right)}^{2}\;\mathrm{for}-H\leq y^{*}\leq 0$$

(20) $$T_{1}{|}_{y^{*}=0}=T_{2}{|}_{y^{*}=0},k_{eff1}{\frac{dT_{1}}{dy^{*}}|}_{y^{*}=0}=k_{eff}{}_{2}{\frac{dT_{2}}{dy^{*}}|}_{y^{*}=0},\;T_{1}{|}_{y^{*}=H}=T_{w},\;T_{2}{|}_{y^{*}=-H}=T_{w}$$

where *T_w_* means the temperature at the channel wall, subscript *i =* 1 implies the conducting nanofluid, and *i =* 2 implies the nonconducting nanofluid. Regarding the thermal properties of power-law nanofluids, the model of Choi and Yu has been applied [30,63] as it is capable of predicting the thermal conductivity of nanoliquids suspended with various kind of nonspherical nanoparticles, namely, (*ρc_p_*)*_effi_*= *ϕ*(*ρc_p_*)*_p_* + (1 − *ϕ*)(*ρc_p_*)*_b_* and $k_{eff}{}_{i}=\frac{k_{p}+2k_{b}{}_{i}+2(k_{p}-k_{b}{}_{i}){\left(1+\omega \right)}^{3}\phi }{k_{p}+2k_{b}{}_{i}-2(k_{p}-k_{b}{}_{i}){\left(1+\omega \right)}^{3}\phi }k_{b}{}_{i}$, where *ω* represents the ratio of nanolayer thickness to the original particle radius and the subscripts *p* and *b* mean nanoparticles and base fluid, respectively. The left-hand side of Equation (18) measures the heat generation due to axial conduction, while the right-hand sides of Equation (18) measure the heat generation caused by heat diffusion, heat generation from Joule heating, and heat generation caused by viscous dissipation. Imposing the constant heat flux boundary condition *q_w_*≡*const* for the fully thermally developed flow above, namely, *∂*[*(T_w_* *−* *T_i_*)/(*T_w_* *−* *T_m_*)]/∂y^*^ = 0, leads to ∂*T*_1_/∂y^*^ = ∂*T*_2_/∂y^*^ = *dT_w_*/dy^*^ = *dT_m_*/dy^*^≡*const*, in which *T_m_* implies the mean temperature [26,55]. Furthermore, the overall energy balance condition over an elemental control volume results in

(21) $$\frac{dT_{m}}{dz^{*}}=\frac{2q_{w}+\lambda HE_{z}{}^{2}+\frac{m_{0}}{{\left(1-\phi \right)}^{5/2}}\left(\int_{0}^{H}|\frac{\partial v_{s1}^{*}}{\partial y_{}^{*}}{|}^{n_{1}-1}\frac{\partial v_{s1}^{*}}{\partial y_{}^{*}}dy^{*}+\int_{-H}^{0}|\frac{\partial v_{s2}^{*}}{\partial y_{}^{*}}{|}^{n_{2}-1}\frac{\partial v_{s2}^{*}}{\partial y_{}^{*}}dy^{*}\right)}{H{\left(\rho c_{p}\right)}_{eff1}v_{ms1}^{*}+H{\left(\rho c_{p}\right)}_{eff2}v_{ms2}^{*}}$$

where $v_{ms1}^{*}=\int_{0}^{H}v_{s1}^{*}dy^{*}/H$ and $v_{ms2}^{*}=\int_{-H}^{0}v_{s2}^{*}dy^{*}/H$ are the dimensional average velocities in layer I and layer II, respectively. Introducing the nondimensional temperature variable *θ_i_* = *k_f_*_1_(*T_i_* − *T_w_*)/(*q_w_H*) and placing Equation (21) into Equations (18)–(20) yields

(22) $$\frac{d^{2}\theta_{1}}{dy^{2}}+\frac{k_{f1}}{k_{eff1}}(S+Br\Phi_{1})=\frac{k_{f1}}{k_{eff1}}\frac{\left(2+S+Br\Gamma_{1}+m_{r}Br\Gamma_{2}\right)}{v_{ms1}+{\left(\rho c_{p}\right)}_{r}v_{ms2}}v_{s1}\;\mathrm{for}\;0\leq y\leq 1$$

(23) $$\frac{d^{2}\theta_{2}}{dy^{2}}+\frac{k_{f1}}{k_{eff2}}m_{r}Br\Phi_{2}=\frac{k_{f1}}{k_{eff2}}\frac{\left(2+S+Br\Gamma_{1}+m_{r}Br\Gamma_{2}\right)}{v_{ms1}/{\left(\rho c_{p}\right)}_{r}+v_{ms2}}v_{s2}\;\mathrm{for}-1\leq y\leq 0$$

(24) $$\theta_{1}{|}_{y=1}=\theta_{2}{|}_{y=-1}=0,\;\theta_{1}{|}_{y=0}=\theta_{2}{|}_{y=0},\;{k_{eff}{}_{1}\frac{d\theta_{1}}{dy}|}_{y=0}=k_{eff}{}_{2}{\frac{d\theta_{2}}{dy}|}_{y=0}$$

where $v_{ms1}=\int_{0}^{1}v_{s1}dy$, $v_{ms2}=\int_{-1}^{0}v_{s2}dy$, $\Phi_{i}=|dv_{si}^{}/dy{|}^{n_{i}-1}{\left(dv_{si}^{}/dy\right)}^{2}$, $\Gamma_{1}=\int_{0}^{1}\Phi_{1}dy$, $\Gamma_{2}=\int_{-1}^{0}\Phi_{2}dy$, (*ρc_p_*)*_r_*= (*ρc_p_*)*_eff_*_2_/(*ρc_p_*)*_eff_*_1_, *m_r_* = *m*_2_/*m*_1_, *Br* = *m*_1_*U*^2^/(*q_w_H*) is the Brinkman number, and *S* = *λHE_z_*^2^/*q_w_* is the Joule heating parameter.

With the two-layer temperature distributions solved from Equations (22)–(24), the heat transfer performance can be examined by evaluating heat transfer rate as represented by the Nusselt number, *Nu = hD_h_/k_eff_.* With the convective heat transfer coefficient at the channel surface *h = q_w_*/(*T_w_ − T_m_*) and the characteristic height *D_h_* = 2*H* [55,57], the further rearrangement produces

(25) $$Nu=-\frac{2k_{f1}}{k_{efff1}\theta_{m}}$$

where $\theta_{m}=(\int_{0}^{1}v_{s1}\theta_{1}dy+\int_{-1}^{0}v_{s2}\theta_{2}dy)/(\int_{0}^{1}v_{s1}dy+\int_{-1}^{0}v_{s2}dy)$ is the mean temperature.

### 2.4. Entropy Generation Analysis

Based on the second law of thermodynamics, a certain amount of energy is inevitably destroyed during the heat transfer process, that is, thermal irreversibility inherently accompanies the thermal behaviors and reduces system efficiency. This irreversibility is represented by the entropy generation rate, and the thermal performance of system is thereby assessed. The local entropy generation over a given cross-section of the microchannel can be given for two-layer flow as

(26) $$S_{l}^{*}{}_{1}(y^{*})=\frac{k_{eff}{}_{1}}{T_{1}{}^{2}}{\left(\frac{dT_{1}}{dy^{*}}\right)}^{2}+\frac{\sigma E_{z}^{2}}{\left|T_{1}\right|}+\frac{m_{eff}{}_{1}}{\left|T_{1}\right|}{\left|\frac{dv_{s1}^{*}}{dy^{*}}\right|}^{n_{i}-1}{\left(\frac{dv_{s1}^{*}}{dy^{*}}\right)}^{2}\;\mathrm{for}\;0\leq y\leq 1$$

(27) $$S_{l}^{*}{}_{2}(y^{*})=\frac{k_{eff}{}_{2}}{T_{2}{}^{2}}{\left(\frac{dT_{2}}{dy^{*}}\right)}^{2}+\frac{m_{eff}{}_{2}}{\left|T_{2}\right|}{\left|\frac{dv_{s2}^{*}}{dy^{*}}\right|}^{n_{2}-1}{\left(\frac{dv_{s2}^{*}}{dy^{*}}\right)}^{2}\;\mathrm{for}-1\leq y\leq 0$$

in which the right-hand side of Equation (26) implies entropy generation caused by heat transfer, Joule heating and viscous dissipation, respectively. With the introduction of the nondimensional groups *S_li_* = H^2^S^*^_li_/*k_f__1_* and Θ = *k_f__1_T_w_*/(*q_w_H*), the respective nondimensional local entropy generation is obtained as follows

(28) $$S_{l}^{}{}_{1}(y)=\frac{k_{eff}{}_{1}}{k_{f1}{\left(\theta_{1}+\Theta \right)}^{2}}{\left(\frac{d\theta_{1}}{dy}\right)}^{2}+\frac{S}{\left|\theta_{1}+\Theta \right|}+\frac{Br}{\left|\theta_{1}+\Theta \right|}{\left|\frac{dv_{s1}}{dy}\right|}^{n_{1}-1}{\left(\frac{dv_{s1}}{dy}\right)}^{2}\;\mathrm{for}\;0\leq y\leq 1$$

(29) $$S_{l}^{}{}_{2}(y)=\frac{k_{eff2}}{k_{f1}{\left(\theta_{2}+\Theta \right)}^{2}}{\left(\frac{d\theta_{2}}{dy}\right)}^{2}+\frac{Br}{\left|\theta_{2}+\Theta \right|}{\left|\frac{dv_{s2}}{dy}\right|}^{n_{2}-1}{\left(\frac{dv_{s2}}{dy}\right)}^{2}\;\mathrm{for}-1\leq y\leq 0$$

The total entropy generation can be computed by integrating Equations (28) and (29) over the relevant cross-section area of microchannel:

(30) $$S_{t}=\int_{0}^{1}S_{l1}dy+\int_{-1}^{0}S_{l2}dy$$

### 2.5. Solutions of Modelling and Validation

#### 2.5.1. In the Case of Newtonian Fluids

The analytical velocities and analytical temperatures of a two-layer transient EOF of Newtonian fluids are first solved, and can then be employed to validate the numerical algorithm proposed for power-law nanofluid flow. Specifically, when *n_i_* = 1 and *ϕ* = 0, Equations (12)–(14) reduce to

(31) $$\frac{\partial v_{1}^{N}}{\partial t}=\frac{\partial^{2}v_{1}^{N}}{\partial y^{2}}-\frac{GE_{z}\zeta }{\cosh(K)}\cosh(Ky)\;\mathrm{for}\;0\leq y\leq 1$$

(32) $$\frac{\partial v_{2}^{N}}{\partial t}=\frac{\partial^{2}v_{2}^{N}}{\partial y^{2}}\;\mathrm{for}-1\leq y\leq 0$$

(33) $$v_{1}^{N}{|}_{y=0}=v_{2}^{N}{|}_{y=0},\;{\frac{\partial v_{1}^{N}}{\partial y}|}_{y=0}={\frac{\partial v_{2}^{N}}{\partial y}|}_{y=0},\;v_{1}^{N}{|}_{y=1}=0,\;v_{1}^{N}{|}_{y=-1}=0,\;v_{1}^{N}{|}_{t=0}=0$$

where the superscript *N* denotes the special case of Newtonian fluid. Using the method of Laplace transformation, the analytical expressions of the transient velocities are obtained as follows:

(34) $$v_{1}^{N}=\frac{GE_{z}\zeta }{K^{2}}\left[\frac{1-\cosh(K)}{2\cosh(K)}(y-1)+\frac{\cosh(Ky)}{\cosh(K)}-1\right]\;+8GE_{z}\zeta \sum_{P=1}^{\infty }\frac{{\left(-1\right)}^{P+1}e^{-{\left(2P-1\right)}^{2}\pi^{2}t/4}}{\left(2P-1)\pi [4K^{2}+{\left(2P-1\right)}^{2}\pi^{2}\right]}\cos\left[\frac{(2P-1)\pi y}{2}\right]\;+GE_{z}\zeta \sum_{P=1}^{\infty }\frac{[1/\cosh(K)-{\left(-1\right)}^{P+1}]e^{-{\left(P\pi \right)}^{2}t}}{P\pi [K^{2}+(P\pi^{2})]}\sin(P\pi y)\;\mathrm{for}\;0\leq y\leq 1$$

(35) $$v_{2}^{N}=\frac{GE_{z}\zeta }{K^{2}}\left[\frac{1-\cosh(K)}{2\cosh(K)}(y-1)+\frac{1}{\cosh(K)}-1\right]\;+8GE_{z}\zeta \sum_{P=1}^{\infty }\frac{{\left(-1\right)}^{P+1}e^{-{\left(2P-1\right)}^{2}\pi^{2}t/4}}{\left(2P-1)\pi [4K^{2}+{\left(2P-1\right)}^{2}\pi^{2}\right]}\cos\left[\frac{(2P-1)\pi y}{2}\right]\;+GE_{z}\zeta \sum_{P=1}^{\infty }\frac{[1/\cosh(K)-{\left(-1\right)}^{P+1}]e^{-{\left(P\pi \right)}^{2}t}}{P\pi [K^{2}+(P\pi^{2})]}\sin(P\pi y)\;\mathrm{for}-1\leq y\leq 0$$

for which the solving procedure is presented in the Appendix A in the interest of conciseness and readability.

As time tends to infinity, the two-fluid velocities reach a steady status as follows:

(36) $$v_{s1}^{N}(y)=\underset{t\to \infty }{\lim}v_{1}^{N}(y,t)=\frac{GE_{z}\zeta }{K^{2}}\left[\frac{1-\cosh(K)}{2\cosh(K)}(y-1)+\frac{\cosh(Ky)}{\cosh(K)}-1\right]\;\mathrm{for}\;0\leq y\leq 1$$

(37) $$v_{s2}^{N}(y)=\underset{t\to \infty }{\lim}v_{2}^{N}(y,t)=\frac{GE_{z}\zeta }{K^{2}}\left[\frac{1-\cosh(K)}{2\cosh(K)}(y-1)+\frac{1}{\cosh(K)}-1\right]\;\mathrm{for}-1\leq y\leq 0$$

which are exactly the solutions to Equations (31)-(33) when the temporal term ∂*v*_i_/∂*t* vanishes.

Replacing Equations (36) and (37) with Equations (22)–(24), the analytical temperature distributions are solved by integrating Equations (22) and (23) twice and combining them with Equation (24):

(38) $$\theta_{1}^{N}=A_{1}y^{3}+A_{2}\cosh(Ky)+A_{3}y^{2}+A_{4}\sinh(Ky)+A_{5}\left[\frac{\cosh{\left(Ky\right)}^{2}}{K^{2}}-y^{2}\right]+D_{1}y+D_{2}$$

(39) $$\theta_{2}^{N}=B_{1}y^{3}+B_{2}y^{2}+D_{3}y+D_{4}$$

with the coefficients *A*, *B*, and *D* presented in the Appendix A for conciseness.

#### 2.5.2. In the Case of Power-Law Nanofluids

Due to the nonlinearity of a two-layer EOF of power-law nanofluids, Equations (12)–(14) and (22)–(24) are numerically solved based on the explicit finite difference method. At first, the following discretization is introduced: $t_{l}=l\Delta t$, $y_{j}=j\Delta y$, $v_{i,j}^{l}=v_{i}(j\Delta y,l\Delta t)$, and $\theta_{i,j}^{l}=\theta_{i}(j\Delta y,l\Delta t)$, *l* = 1, 2, …, *L* and *j* = 1, 2, …, *J*.

At first, the numerical velocities at the channel walls are easily determined by discretizing the no slip conditions in Equation (14):

(40) $$v_{1,J}^{l}=v_{2,1}^{l}=0$$

The numerical velocities at the two-liquid interface are computed using the bisection method from the discretized version of the two-liquid interface matching conditions in Equation (14)

(41) $$v_{1,1}^{l}=v_{2,J}^{l},\;m_{1}{\left(\frac{-3v_{1,1}^{l}+4v_{1,2}^{l}-v_{1,3}^{l}}{2\Delta y}\right)}^{n_{1}}=m_{2}{\left(\frac{v_{2,J-2}^{l}-4v_{2,J-1}^{l}+3v_{2,J}^{l}}{2\Delta y}\right)}^{n_{2}}$$

Note that for the proposed numerical algorithm, when *n_i_* ≥ 1, let the initial two-layer velocities be $v_{1}^{0}=v_{2}^{0}=0$, and when *n_i_* < 1, to avoid the singularity caused by the zero denominator, let the initial two-layer velocities be $v_{1}^{0}=v_{2}^{0}=nonzero$.

Then, the velocities of the bulk liquid can be computed by means of the following numerical algorithm:

(42) $$v_{1,j}^{l+1}=v_{1,j}^{l}+[\Lambda_{1,j}^{l}+GE_{z}\zeta \cosh(Ky_{j})/\cosh(K)]\cdot \Delta t$$

(43) $$v_{2,j}^{l+1}=v_{2,j}^{l}+\Lambda_{2,j}^{l}\cdot \Delta t$$

where $\Lambda_{i,j}^{l}=\frac{m_{i}}{m_{0}}\left[{\left(g_{i,j}^{l}\right)}^{n_{i}-1}\frac{v_{i,j+1}^{l}-2v_{i,j}^{l}+v_{i,j-1}^{l}}{\Delta y^{2}}+(n_{i}-1){\left(g_{i,j}^{l}\right)}^{n_{i}-2}\frac{g_{i,j+1}^{l}-g_{i,j-1}^{l}}{2\Delta y}\frac{v_{i,j+1}^{l}-v_{i,j-1}^{l}}{2\Delta y}\right]$, $g_{i,j}^{l}=\left|\frac{v_{i,j+1}^{l}-v_{i,j-1}^{l}}{2\Delta y}\right|$, *i* = 1, 2, and *j* = 2, 3, …, *J* − 1. When *t* is great enough, the transient velocities reach steady status, i.e., they are examined by $\left\|v^{l}-v^{l+1}\right\|<Er$, with *Er* being a specified criterion, and the steady velocities *v_s_*_1_ and *v_s_*_2_ are solved numerically. Consequently, the flow rates can be computed from Equations (15) and (16) by means of the Simpson composite integration method [61].

The temporal term *∂θ_i_*/*∂t* is introduced to Equations (22) and (23) to allow the same numerical algorithm to be used to solve both velocity distribution and temperature distribution. As time approaches to infinity, the fully thermally developed temperature distribution can be obtained. More specifically, at first the numerical temperatures at the boundaries and initial condition can be easily determined from

(44) $$\theta_{1,J}^{l}=\theta_{2,1}^{l}=0,\;\theta_{1}^{0}=\theta_{2}^{0}=0$$

(45) $$\theta_{1,1}^{l}=\theta_{2,J}^{l},\;k_{eff}{}_{1}\frac{-3\theta_{1,1}^{l}+4\theta_{1,2}^{l}-\theta_{1,3}^{l}}{2\Delta y}=k_{eff2}\frac{\theta_{2,J-2}^{l}-4\theta_{2,J-1}^{l}+3\theta_{2,J}^{l}}{2\Delta y}$$

Then, the temperature distributions of the bulk liquid are computed based on the following numerical algorithm:

(46) $$\theta_{1,j}^{l+1}=\theta_{1,j}^{l+1}+(\Pi_{1,j}^{l}+k_{f1}S/k_{eff1})\cdot \Delta t$$

(47) $$\theta_{2,j}^{l+1}=\theta_{2,j}^{l}+(\Pi_{2,j}^{l})\cdot \Delta t$$

where $\Pi_{i,j}^{l}=\left[\frac{\theta_{i,j+1}^{l}-2\theta_{i,j}^{l}+\theta_{i,j-1}^{l}}{\Delta y^{2}}+a_{i}\frac{k_{f1}}{k_{eff}{}_{i}}\Phi_{i,j}^{l}-\frac{k_{f1}}{k_{eff}{}_{i}}\frac{2+S+Br\Gamma_{1}^{l}+m_{r}Br\Gamma_{2}^{l}}{b_{i}v_{ms1}^{l}+c_{i}v_{ms2}^{l}}v_{si}^{l}\right]$, $\Phi_{i,j}^{l}={\left(g_{i,j}^{l}\right)}^{n_{i}-1}{\left(\frac{v_{si,j+1}^{l}-v_{si,j-1}^{l}}{2\Delta y}\right)}^{2}$, $a_{1}=b_{1}=c_{2}=1$, $a_{2}=m_{r}$, $b_{2}=1/{\left(\rho c_{p}\right)}_{r}$, $c_{1}={\left(\rho c_{p}\right)}_{r}$, *i* = 1, 2, *j* = 2, 3, …, *J –* 1, and the integrals $\Gamma_{1}^{l}=\int_{0}^{1}\Phi_{1}^{l}dy$, $\Gamma_{2}^{l}=\int_{-1}^{0}\Phi_{2}^{l}dy$, $v_{ms}^{l}{}_{1}=\int_{0}^{1}v_{s1}^{l}dy$, $v_{ms}^{l}{}_{2}=\int_{-1}^{0}v_{s2}^{l}dy$ are computed using the Simpson composite integration method. As time grows, if $\left\|\theta^{l}-\theta^{l+1}\right\|<Er$, the thermally fully developed numerical temperature distribution is obtained, based on which the Nusselt number in Equation (25) and total entropy generation in Equation (30) can be computed and the heat transfer analysis is conducted.

The physical parameters regarding the hydrodynamic properties of two power-law nanofluids are: *m*_0_^*^ = 9 × 10^−4^ Nm^−2^*s^n^*, *H* = 1 × 10^−^^4^ m, and *U* = 1 × 10^−4^ m·s^−^^1^, which is of the same order as the Helmholtz–Smoluchowski velocity [52,53,61]. The physical parameters regarding the electric properties of two power-law nanofluids are : *ε*_2_ = 7.08 × 10^−10^ F/m, *e* = 1.6 × 10^−19^ C, *z*_0_ = 1, E_z_^*^ = 1 × 10^4^ V·m^−^^1^, *k_B_* = 1.38 × 10^−23^ J·K^−1^, and ζ^*^ = −0.025 V [61]. To facilitate discussion, let the thermophysical properties of the fluids remain constant; the thermal conductivity of two base fluids are *k_b_*_1_ = *k_b_*_2_ = 0.618 Wm^−1^K^−1^, the thermal conductivity of AI_2_O_3_ is *k_p_* = 40 Wm^−1^K^−1^, *T*_0_ = 293 K, andthe Joule heating parameter *S* = 1 [61]. Furthermore, it is essential to provide the ranges of important nondimensional governing parameters based on practical physical uses. From the well-established electroosmotic theory of power-law modeling, *n_i_* = 0.6~1.4 [23] and the width of EDL is far less than the channel height; thus, *K* = 10~100 [23,52]. Based on the given order of reference (velocity, viscosity, and channel height), the Brinkman number ranges from *Br* = 0~0.1 [31,55], and the nanoparticle volume fraction *ϕ* = 0~0.09 is suitable for the chosen model of effective viscosity and effective thermal conductivity [44].

**Figure 2:**
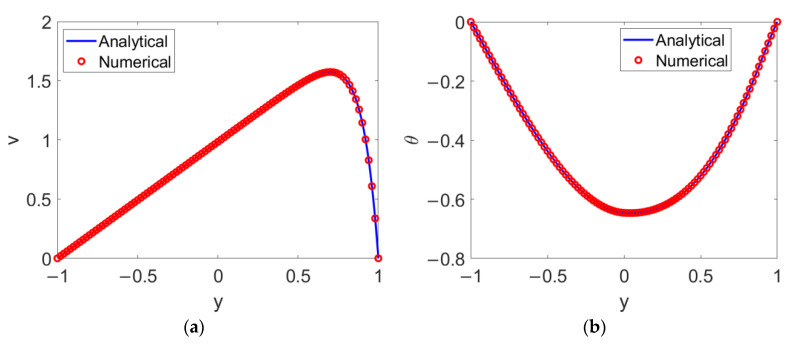
Comparison of analytical solutions and numerical solutions for (**a**) velocity and (**b**) temperature.

Concerning the numerical schemes, let *Er* = 1 × 10^−^^8^ in order to assure that the velocity and temperature reach steady status; then, a test grid dependence is conducted, with a grid system of 1 × 10^3^ chosen. The good agreement between the analytical solutions and numerical solutions depicted in Figure 2 shows that the numerical algorithm proposed here is feasible for computing the two-layer velocity distribution and two-layer temperature distribution and for carrying out the heat transfer analysis.

## 3. Results and Discussion

The transient hydrodynamic behavior of two-layer power-law nanofluid EOF is discussed by evaluating the transient two-layer velocities at different times and the flow rates for different governing nondimensional parameters. Then, with the steady velocities computed, the heat transfer analysis and entropy generation analysis are conducted by presenting the two-layer temperature profiles, Nusselt number and entropy generation rate at different governing nondimensional parameters. Specifically, *(ρC_p_*)*_r_* = 1 and *ρ_r_* = 1, such that their importance can be attached to the effects of power-law rheology (represented by *n_i_*), the electroosmotic property (represented by electrokinetic width *K*), the nanoparticle volume fraction, *ϕ*, and the viscous dissipation effect (represented by the Brinkman number, *Br*) on hydrodynamic and thermal behaviors.

### 3.1. Flow Characteristics in Two-Layer

**Figure 3:**
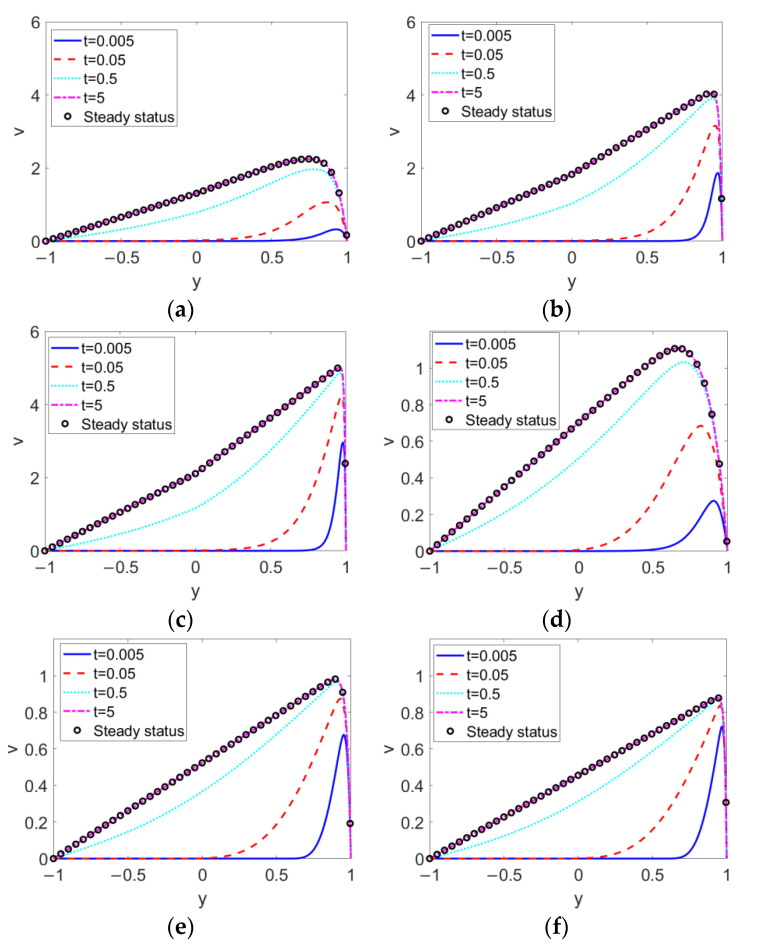
Transient two-layer velocities at different electrokinetic widths *K* and different flow behavior indexes of conducting fluid *n*_1_ when *Br* = 0.02, *S* = 1, *ζ* = −1, *n*_2_ = 1.2, and *ϕ* = 0.03. (**a**) *K* = 10, *n*_1_ = 0.8; (**b**) *K* = 50, *n*_1_ = 0.8; (**c**) *K* = 100, *n*_1_ = 0.8; (**d**) *K* = 10, *n*_1_ = 1.2; (**e**) *K* = 50, *n*_1_ = 1.2; (**f**) *K* = 100, *n*_1_ = 1.2.

The time evolutions of two-layer velocities with different types of conducting nanofluid, i.e., flow behavior index *n*_1_ at different electrokinetic width *K*, are presented in Figure 3. It can be seen that at first the conducting nanofluid near the channel wall is set in motion due to the electroosmotic force, and as time elapses the bulk conducting nanofluid attains velocity. As opposed to the single-layer EOF, the flow of bulk conducting fluid fails to form a plug-like profile, as the delivery of momentum via the bulk conducting nanofluid is dissipated through interfacial viscous stress, causing a deviated parabolic profile in layer I. Accordingly, the nonconducting nanofluid is driven by interfacial shear stress; thus, in layer II, the closer to the two-liquid interface the flow, the higher the velocity. No matter what value *K* or *n*_1_ takes, the conducting nanofluid near the wall at first moves forward and drags the nonconducting fluid through the interfacial hydrodynamical shear stress, and as *t* increases to 5, the transient two-layer velocity reaches its steady state. The comparison among Figure 3a–c describes the way in which, when the conducting nanofluid is shear thinning, the transient two-layer velocity is augmented with the increase of *K*, while as shown in Figure 3d–f, when the conducting nanofluid is shear thickening, the transient two-layer velocity shows abatement with the increase of *K*. The profiles of the steady velocities in Figure 3b,c show that the remarkable increase in the velocity of the conducting nanofluid with *K* results in a subtle turning of the velocity near the two-liquid interface. Moreover, when pumped by shear thinning nanofluid, the two-layer fluid is more sensitive to changes in the electrokinetic width *K* than when pumped by shear-thickening nanofluid.

**Figure 4:**
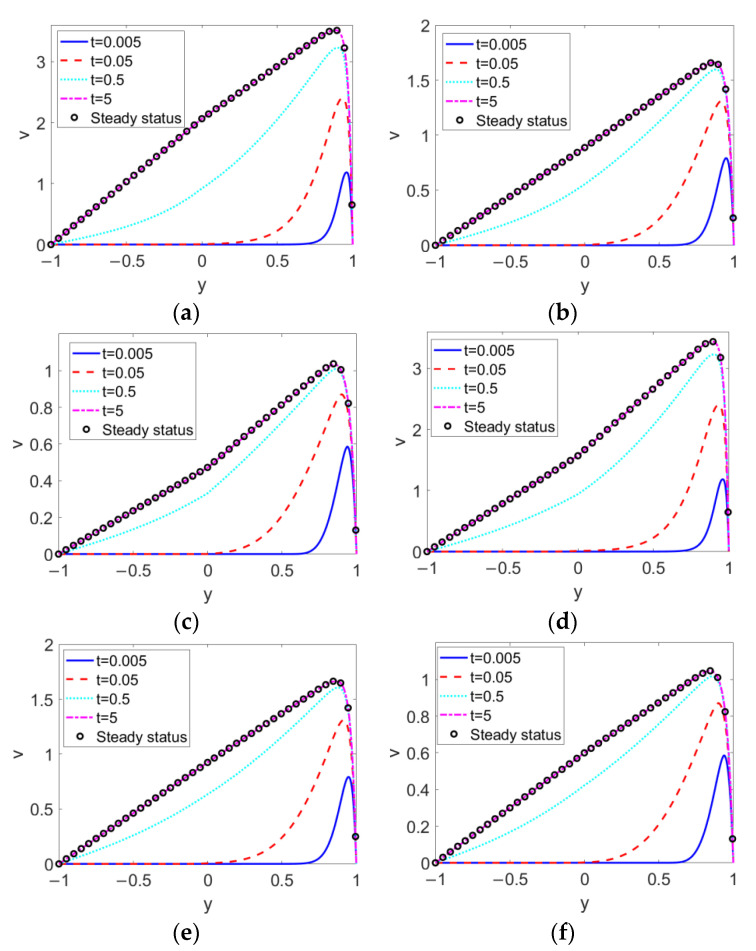
Transient two-layer velocities at different flow behavior indexes *n*_1_ and different flow behavior indexes *n*_2_ when *Br* = 0.02, *S* = 1, *ζ* = −1, *K* = 30 and *ϕ* = 0.03. (**a**) *n*_1_ = 0.8, *n*_2_ = 0.6; (**b**) *n*_1_ = 1, *n*_2_ = 0.6; (**c**) *n*_1_ = 1.2, *n*_2_ = 0.6; (**d**) *n*_1_ = 0.8, *n*_2_ = 1.4; (**e**) *n*_1_ = 1, *n*_2_ = 1.4; (**f**) *n*_1_ = 1.2, *n*_2_ = 1.4.

When the conducting nanofluid and nonconducting nanofluid change from shear thinning to shear thickening, the time evolutions of the two-layer velocities are as delineated in Figure 4. No matter what type of pumped nonconducting nanofluid is considered, the decrease in the flow behavior index *n*_1_, namely, the shear thinning feature of conducting fluid, accelerates the flow and consequently enhances the two-layer velocity. From the comparison between Figure 4a–e, the change in nonconducting nanofluid type from shear thinning to shear thickening exerts a slight influence on the two-layer flow near the two-liquid interface, and shows little influence on that far away from the two-liquid interface. The influence of *n*_1_ on two-layer flow far outweighs that of *n*_2_ on two-layer flow.

**Figure 5:**
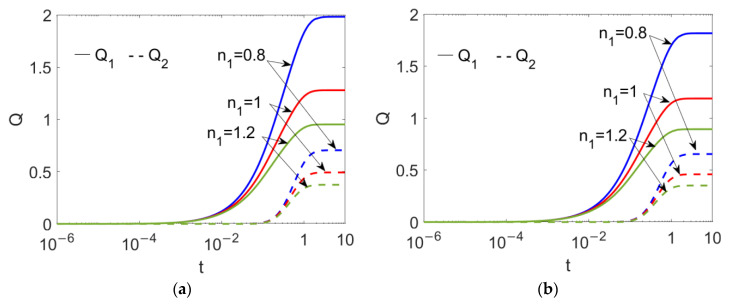
Time evolutions of flow rates of two power-law nanofluids at different flow behavior indexes *n*_1_ and different volume fractions of nanoparticle *ϕ* when *Br* = 0.02, *S* = 1, *ζ* = −1, *K* = 10 and *n*_2_ = 1.2. (**a**) *ϕ* = 0; (**b**) *ϕ* = 0.03.

Because the flow behavior index, *n*_1_, plays a crucial role in two-layer velocity, the time evolutions of flow rates at different flow behavior indexes of conducting nanofluid *n*_1_ are presented in Figure 5 when (a) *ϕ* = 0 and (b) *ϕ* = 0.03. It is obvious that the unsteady flow rate of conducting nanofluid takes a longer time to reach steady status than that of nonconducting nanofluid; as the conducting nanofluid near the wall is set in motion first, the bulk conducting nanofluid moves forward, and eventually the nonconducting nanofluid is dragged by the bulk conducting fluid. Irrespective of the value of the nanoparticle volume fraction *ϕ*, the shear thinning feature of the conducting fluid enhances the flow rates of both the conducting and nonconducting nanofluids. A comparison between Figure 5a,b shows that the addition of the nanoparticle to the bulk nanofluid reduces the two-layer flow rate by improving the viscosity of the two fluids, as per Equations (9) and (10).

**Figure 6:**
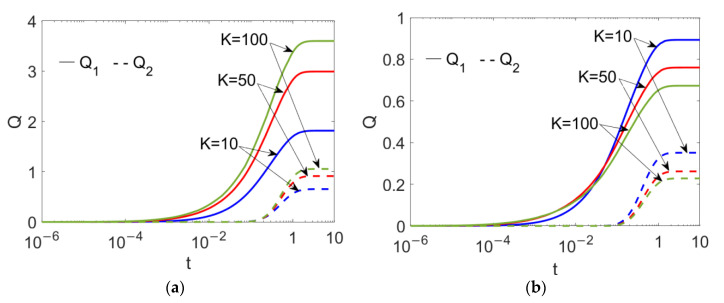
Time evolutions in flow rates of two power-law nanofluids at different flow behavior indexes *n*_1_ and different electrokinetic widths *K* when *Br* = 0.02, *S* = 1, *ζ* = −1, *n*_2_ = 1.2 and *ϕ* = 0.03. (**a**) *n*_1_ = 0.8; (**b**) *n*_1_ = 1.2.

In Figure 6, the time evolutions of flow rates at different electrokinetic widths *K* are presented when (a) *n*_1_ = 0.8 and (b) *n*_2_ = 1.2. From Figure 6a, when the pumping conducting nanofluid is shear thinning, the flow rates of the two power-law nanofluids show augmentation with the electrokinetic width *K*, being consistent with Figure 3; in contrast, when it is shear thickening, the flow rates of both fluids show abatement with *K*, meaning that the change in fluid type of the pumping nanofluid from shear thinning to shear thickening can reverse the effect of the electrokinetic width. In addition, the flow rates show a noticeable reduction when *n*_1_ increases, irrespective of what value *K* takes.

**Figure 7:**
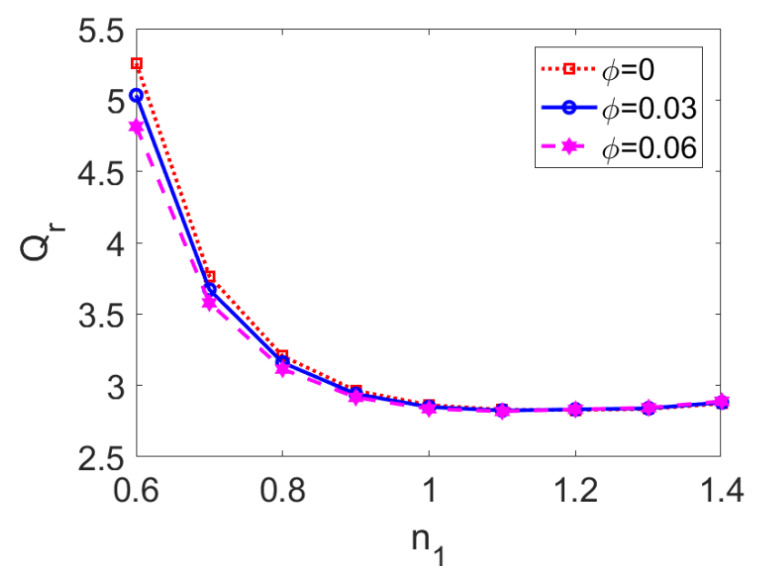
Variation of the ratio of flow rates with flow behavior index *n*_1_ at different nanoparticle volume fractions *ϕ* when *Br* = 0.02, *S* = 1, *ζ* = −1, *n*_2_ = 1.2, and *K* = 30.

In Figure 7, the variation in the ratio of flow rate I to flow rate II is plotted for a flow behavior index of conducting nanofluid *n*_1_ at different nanoparticle volume fractions *ϕ*. When the pumping conducting nanofluid is shear thinning, that is, *n*_1_ < 1, flow rate I is more than three times higher than flow rate II and two-layer flow is evidently affected by the change in the nanoparticle volume fraction, *ϕ*. In contrast, when the pumping conducting nanofluid is shear thickening (i.e., *n*_1_ > 1), the two-layer flow is insensitive to the change in flow behavior index *n*_1_ and nanoparticle volume fraction *ϕ*. Compared to the effect of the nanoparticle volume fraction, the effect of *n*_1_ dominates in the two-layer flow.

**Figure 8:**
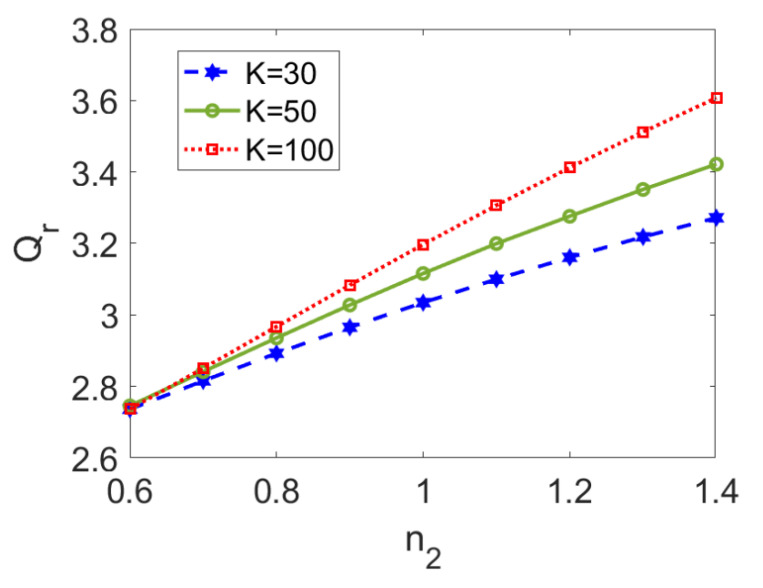
Variation of the ratio of volumetric flow rates with flow behavior index *n*_2_ at different nanoparticle volume fractions when *Br* = 0.02, *S* = 1, *ζ* = −1, *n*_1_ = 0.8, and *ϕ* = 0.03.

In Figure 8, the variation in the ratio of flow rate I to flow rate II is presented for the flow behavior index of nonconducting nanofluid *n*_2_ at different electrokinetic widths *K*. A higher flow behavior index of nonconducting nanofluid triggers stronger viscosity, and thus smaller flow rate of nonconducting nanofluid flow, finally leading to an increase in the flow rate ratio. When the type of conducting nanofluid is fixed, although a thinner EDL length improves velocities of both fluids (as shown in Figure 3a–c), the increase in flow rate I is much greater than that of flow rate II, and thus the increasing trend of flow rate ratio with *K* is observed, which is especially obvious for a shear thickening nonconducting nanofluid. Therefore, when dragging a shear thickening fluid, increasing electrokinetic width exerts more influence on flow rate I than on flow rate II. Furthermore, in comparison with Figure 7, the effect of the flow behavior index, *n*_2_, on the flow rate ratio is almost linear, and the increasing rate is enhanced for greater values of the electrokinetic width, *K*.

### 3.2. Heat Transfer Characteristics in Two-Layer Flow

**Figure 9:**
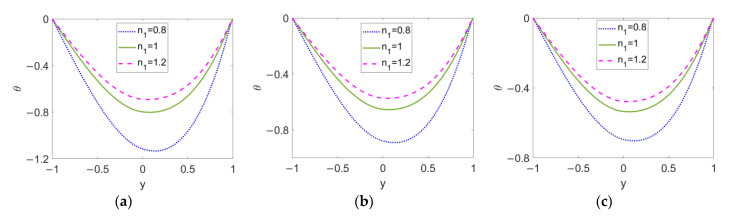
Variation of temperature at different flow behavior indexes *n*_1_ and different nanoparticle volume fractions *ϕ* when *K* = 30, *n*_2_ = 1.2, *ζ* = −1, *S* = 1, and *Br* = 0.02. (**a**) *ϕ* = 0; (**b**) *ϕ* = 0.03; (**c**) *ϕ* = 0.06.

In Figure 9, the temperature profiles at different flow behavior indexes *n*_1_ are described when (a) *ϕ* = 0, (b) *ϕ* = 0.03, and (c) *ϕ* = 0.06. It is noted that the temperature profiles of the two-layer flow are asymmetric around the two-liquid interface, in which the minimum value occurs within layer I. The temperature difference between the channel wall and bulk fluid is reduced, with the conducting nanofluid changing from a shear thinning fluid to a shear thickening one; therefore, it can be seen that the smaller the velocity gradient of two-layer flow is, the better the heat transfer performance becomes. On the other hand, the increase in the nanoparticle volume fraction, *ϕ*, leads to an overall increase in the temperature profiles for all types of conducting nanofluid, meaning that the introduction of nanoparticles truly improves the heat transfer in two-layer flow.

**Figure 10:**
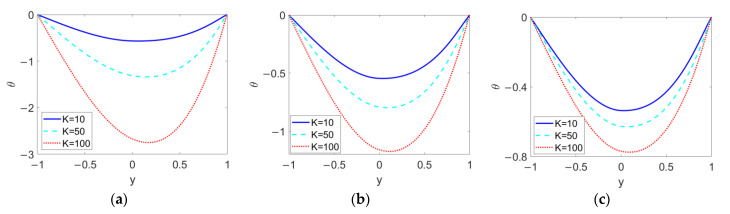
Variation of temperature at different flow behavior indexes *n*_1_ and different electrokinetic widths *K* when *ϕ* = 0.03, *n*_2_ = 1.2, *ζ* = −1, *S* = 1, and *Br* = 0.02. (**a**) *n*_1_ = 0.8; (**b**) *n*_1_ = 1; (**c**) *n*_1_ = 1.2.

As shown in Figure 10, the temperature profiles at different electrokinetic widths *K* are plotted when (a) *n*_1_ = 0.8, (b) *n*_1_ = 1, and (c) *n*_1_ = 1.2 in order to demonstrate the influence of *n*_1_ on the effect of *K*. The increase in *K*, namely a thinner EDL thickness, enlarges the temperature difference between the channel wall and bulk liquid and shifts the minimum value of the temperature to the right. This can be explained by the fact that the increase in *K* enhances the two-layer velocity and causes a drastic change in velocity near the channel wall, as described in Figure 3. The comparison among Figure 10a–c indicates that the shear thickening feature of the conducting nanofluid tends to suppress the effect of the electrokinetic width, *K*, on temperature profile.

**Figure 11:**
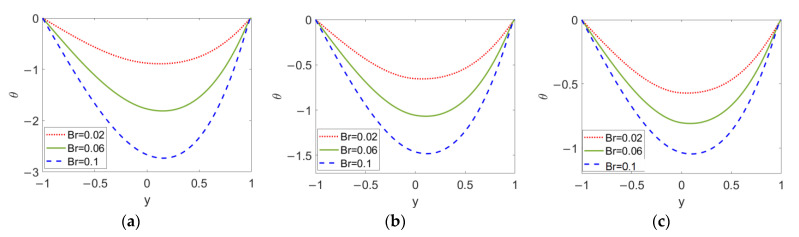
Variation of temperature at different flow behavior indicese *n*_1_ and different Brinkman numbers *Br* when *ϕ* = 0.03, *n*_2_ = 1.2, *ζ* = −1, *S* = 1 and, *K* = 30. (**a**) *n*_1_ = 0.8; (**b**) *n*_1_ = 1; (**c**) *n*_1_ = 1.2.

As shown in Figure 11, the temperature profiles with different Brinkman numbers *Br* are plotted when (a) *n*_1_ = 0.8, (b) *n*_1_ = 1, and (c) *n*_1_ = 1.2 in order to show the combined effect of flow behavior index *n*_1_ and Brinkman number *Br*. As the Brinkman number *Br* increases, viscous dissipation in the two-layer flow is improved, which further enlarges the temperature difference between the channel wall and bulk liquid, meaning that the consideration of viscous dissipation evidently hinders the heat transfer in two-layer flow. Moreover, as obvious changes in temperature are observed in Figure 11a–c, the Brinkman number *Br* has an important effect on the temperature distribution for all types of conducting nanofluid; in other word, no matter what type of fluid the two-layer flow is driven by, the effect of *Br* on temperature cannot be neglected.

**Figure 12:**
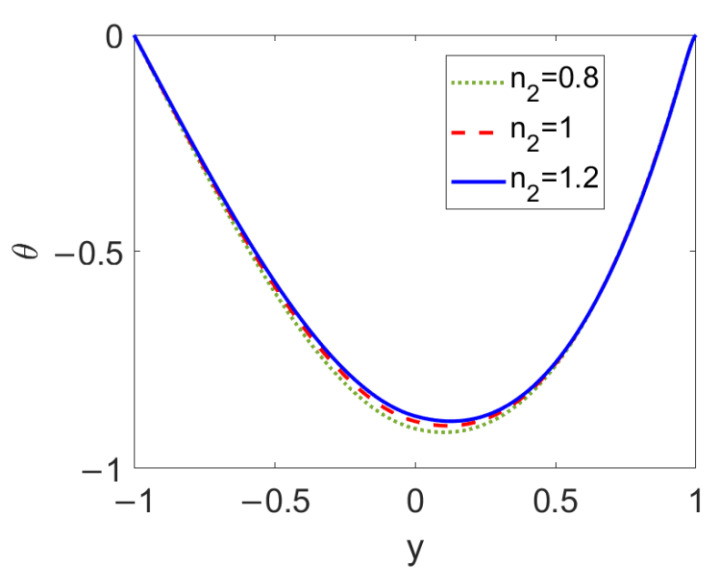
Variation of temperature at different flow behavior indexes *n*_2_ when *ϕ* = 0.03, *n*_1_ = 0.8, *ζ* = −1, *S* = 1, and *Br* = 0.02.

The temperature profiles at different flow behavior indices of nonconducting nanofluid *n*_2_ are described in Figure 12. When the pumped nonconducting nanofluid changes from a shear thinning fluid to a shear thickening one, the temperature in the vicinity of the two-liquid interface slightly increases, while far away from the two-liquid interface it shows little change. This is because the increase in the flow behavior index, *n*_2_, causes a slight change in the two-layer velocity near the two-liquid interface. Therefore, compared to the influence of the fluid type of the pumping conducting nanofluid, that of the fluid type of the pumped nonconducting nanofluid (*n*_2_) on temperature profile is weak.

**Figure 13:**
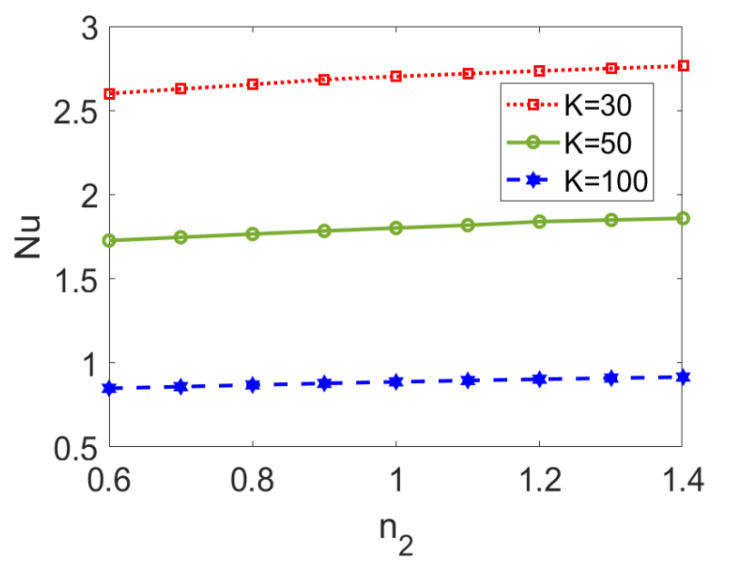
Variation of Nusselt number with flow behavior index *n*_2_ at different electrokinetic widths *K* when *ϕ* = 0.03, *n*_1_ = 0.8, *ζ* = −1, *S* = 1, and *Br* = 0.02.

The variation in the Nusselt number *Nu* with flow behavior index *n*_2_ at different electrokinetic widths *K* is presented in Figure 13. The Nusselt number, *Nu*, shows a slightly ascending trend with flow behavior index *n*_2_ no matter the value of the electrokinetic width. On the other hand, the increase in electrokinetic width, *K*, leads to the decrease in Nusselt number, *Nu.* This is because the rapid change in velocity profile caused by a lower EDL thickness triggers a widened temperature difference by suppressing the heat transfer performance of the two-layer flow. It should be noted that the effect of electroosmotic property of the fluid and the effect of the type of pumped nonconducting nanofluid do not interact with each other.

**Figure 14:**
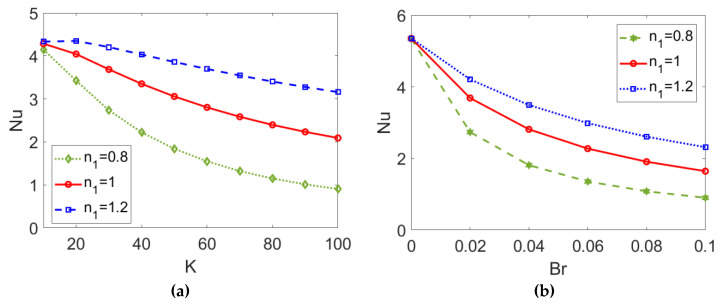
Variation of the Nusselt number *Nu* with (a) electrokinetic width *K* and (b) Brinkman number *Br* at different flow behavior indexes *n*_1_ when *ϕ* = 0.03, *ζ* = −1, *n*_2_ = 1.2, and *S* = 1. (**a**) *Br* = 0.02; (**b**) *K* = 30.

In Figure 14, the variation of the Nusselt number *Nu* with (a) electrokinetic width *K* and (b) Brinkman number *Br* is presented when choosing different types of conducting nanofluid. As shown in Figure 14a, a greater value of the electrokinetic width *K* reduces the heat transfer in two-layer flow, causing the dramatic change in velocity and widening temperature difference shown in Figure 3 and Figure 10. Figure 14b shows that the Nusselt number *Nu* decreases with the Brinkman number *Br*, as the stronger viscous dissipation effect represented by greater values of *Br* enlarges the temperature difference between the channel wall and the bulk liquid, impeding heat transfer in two-layer flow. As opposed to the interaction between the effects of *K* and *n*_2_ predicted in Figure 13, the descending trend of the Nusselt number *Nu* with *K* or *Br* becomes smaller when the conducting nanofluid changes from a shear thinning fluid to a shear thickening one, meaning that when two-layer flow is driven by a shear thinning fluid the heat transfer performance is much more susceptible to changes in electrokinetic width, *K*, or the Brinkman number, *Br*.

**Figure 15:**
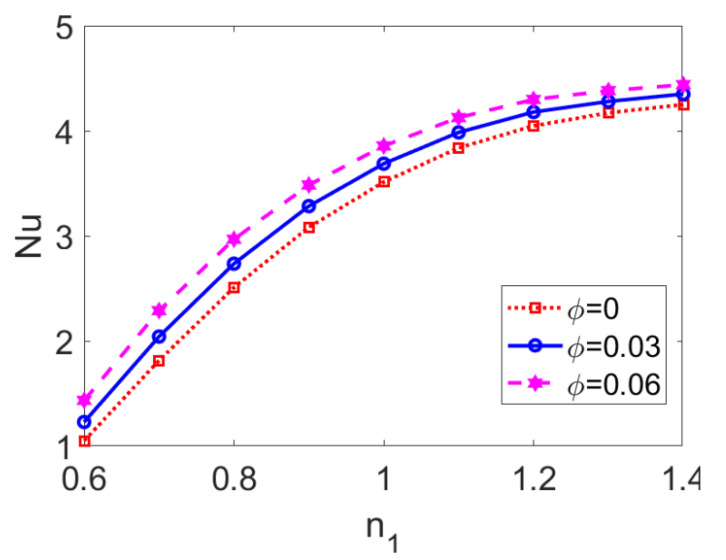
Variation of Nusselt number *Nu* with flow behavior index *n*_1_ at different volume fractions of nanoparticles *ϕ* when *K* = 30, *n*_2_ = 1.2, *ζ* = −1, *S* = 1, and *Br* = 0.02.

Variation of the Nusselt number *Nu* with flow behavior index *n*_1_ at different nanoparticle volume fractions *ϕ* is shown in Figure 15, revealing the interaction between the effect of the conducting nanofluid and that of the nanoparticles. As the conducting nanofluid changes from a shear thinning fluid to a shear thickening one, the Nusselt number *Nu* is augmented, hence the heat transfer performance of two-layer flow is enhanced. Furthermore, the ascending trend of *Nu* with *n*_1_ becomes more slight. The increment in the nanoparticle volume fraction *ϕ* tends to improve the variation of *Nu* with *n*_1_ as a whole, thus promoting the heat transfer of two-layer flow for all types of conducting nanofluid. This means that the addition of nanoparticles has little influence on the effect of the conducting nanofluid type on heat transfer (represented by *Nu*). According to Equation (25), growth in mean temperature with *ϕ* leads to the increase in *Nu*, implying that the temperature difference between the bulk fluid and channel walls is narrowed. Therefore, in practical engineering terms, when a wide temperature difference between the channel wall and the centerline occurs and might cause undesirable results (such as electrochemical decomposition or fluctuation of PH value in working liquids), the introduction of nanoparticles can intensify heat transfer performance and help to avoid the problems mentioned above.

**Figure 16:**
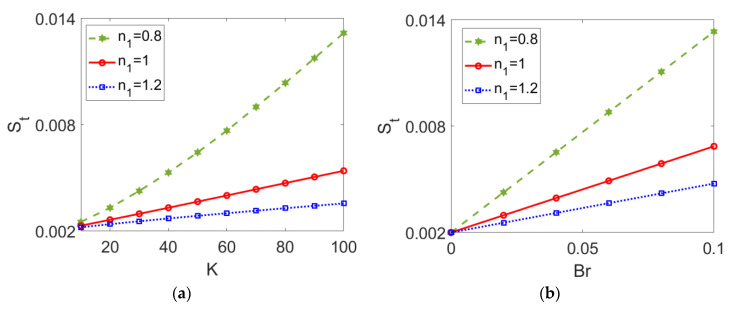
Variation of total entropy generation with (**a**) electrokinetic width *K* and (**b**) Brinkman number *Br* at different flow behavior indexes *n*_1_ when *ϕ* = 0.03, *n*_2_ = 1.2, and *S* = 1. (**a**) *Br* = 0.02; (**b**) *K* = 30.

The variation of total entropy generation *S_t_* with (a) electrokinetic width *K* and (b) Brinkman number *Br* is presented in Figure 16 for different types of conducting nanofluids. An almost linear increase of total entropy generation *S_t_* with electrokinetic width *K* and Brinkman number *Br* can be observed in Figure 16. From Figure 16a, it can be inferred that a thinner EDL length (represented by a greater value of *K*) results in a wider velocity gap between the channel wall and the bulk fluid; therefore, the heat transfer is retarded, and entropy generation improves accordingly. Figure 16b implies that the viscous dissipation effect grows stronger with the Brinkman number, leading to an increase in entropy generation. In addition, the increasing trend with *K* and *Br* becomes smaller when nanofluid I changes from shear thinning to shear thickening.

**Figure 17:**
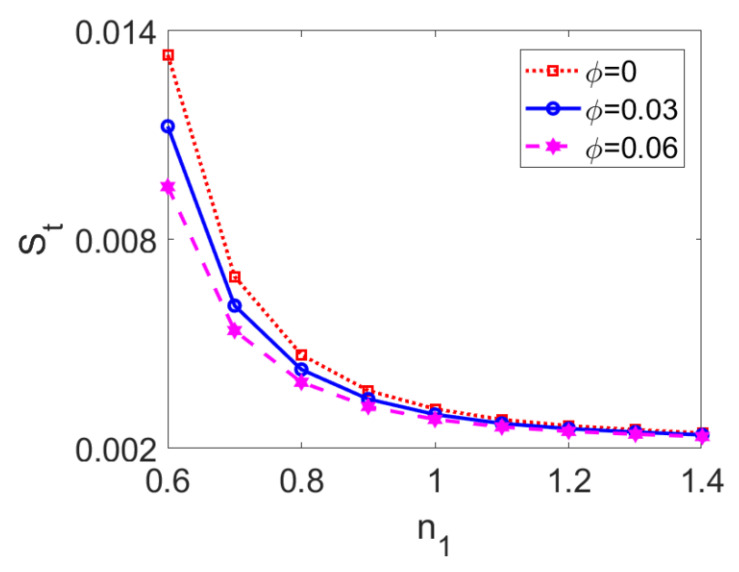
Variation of total entropy generation with flow behavior index *n*_1_ at different nanoparticle volume fractions *ϕ* when *K* = 30, *n*_2_ = 1.2, *S* = 1, and *Br* = 0.02.

The variation of total entropy generation with flow behavior index *n*_1_ at different nanoparticle volume fractions *ϕ* is presented in Figure 17. The entropy generation decreases when the conducting nanofluid changes from a shear thinning type to a shear thickening one. The shear thinning feature of conducting fluid accelerates the two-layer flow and enhances velocity distribution, while the increased velocity gradient and temperature gradient result in greater entropy generation as per Equations (28) and (29). Furthermore, entropy generation in two-layer flow driven by shear thinning nanofluid is more sensitive to changes in nanoparticle volume fraction compared to that driven by shear thickening nanofluid. In practical terms, when the two-layer flow is pumped by shear thinning fluid, it is more likely to utilize nanoparticles to adjust the thermal performance of the system.

## 4. Conclusions

(1) The hydrodynamic behavior of transient two-layer EOFs of power-law nanofluids in a slit microchannel were investigated by evaluating the transient two-layer velocity distribution at different times and with different two-layer flow rates.
  - When driven by shear thinning nanofluid, the two-layer flow accelerates for thinner EDL thicknesses and decelerates when driven by shear thickening nanofluid. The change in fluid type of pumped nonconducting nanofluid exerts only a slight influence on velocity near the two-liquid interface. It is concluded that compared to the fluid type of pumped nonconducting nanofluid, the fluid type of the pumping conducting nanofluid plays a dominant role in two-layer flow and alters the effect of the electrokinetic width, *K*.
  - In practical terms, the selection of a conducting nanofluid is crucial, as is the use of electrokinetic width to adjust two-layer flow for different types of conducting nanofluid.
  - As opposed to the variation of the flow rate ratio with *n*_2_, the variation with *n*_1_ is nonlinear, and the flow rate of two-layer flow driven by shear thinning nanofluid is more sensitive to changes in the nanoparticle volume fraction.
(2) With steady two-layer velocity obtained, the thermally developed heat transfer characteristics were discussed by presenting the temperature distribution, Nusselt number, and total entropy generation at different parameters.
  - The fluid type of the pumping conducting nanofluid, Brinkman number, nanoparticle volume fraction, and electrokinetic width all play important roles in the temperature profile, Nusselt number, and total entropy generation; in contrast, the influence of the type of pumped nonconducting nanofluid is weak.
  - In terms of the interactive influence of the governing parameters, shear thickening feature of the conducting nanofluid tends to suppress the effects of the Brinkman number and electrokinetic width on heat transfer and entropy generation.
  - No matter what type of conducting nanofluid is considered, increasing the nanoparticle volume fraction within a specified range truly enhances the heat transfer performance of two-layer flow.
  - Entropy generation in two-layer flow driven by shear thinning nanofluid is more sensitive to changes in electrokinetic width, Brinkman number, and nanoparticle volume fraction.

## Figures and Tables

**Figure A1:**
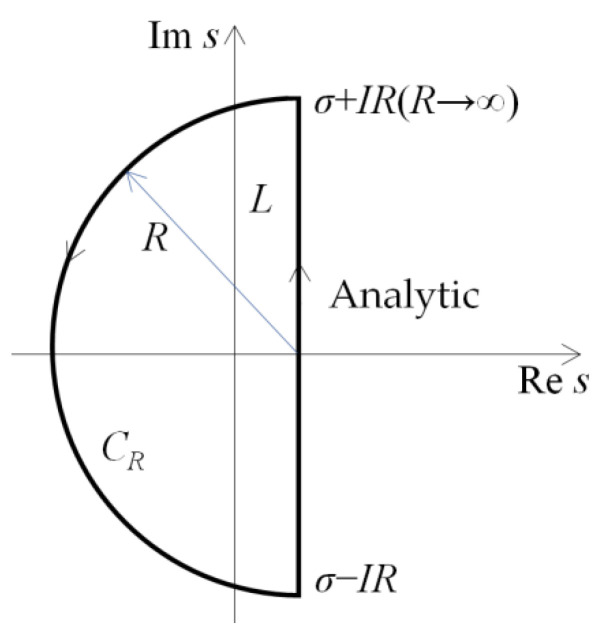
Schematic of the simple closed curve *C_R_* + *L* in the *s*-plane.

## Data Availability

Not applicable.

## Appendix A

To obtain the analytical solutions for two-layer EOF in the case of Newtonian fluid, Laplace transform to two-layer velocity distributions *v_i_* is introduced:

(A1) $$V_{i}(s)=\text{ℒ}\left[v_{i}(t)\right]=\int_{0}^{\infty }v_{i}^{N}(y,t)e^{-st}dt$$

Transforming Equations (31)–(33), and letting *ρ_r_* = 1, the ordinary differential equations (ODEs) with respect to *s* can be obtained as follows:

(A2) $$\frac{d^{2}V_{1}}{dy^{2}}-sV_{1}=\frac{1}{s}GE_{z}\zeta \frac{\cosh(Ky)}{\cosh(K)}\;\mathrm{for}\;0\leq y\leq 1$$

(A3) $$\frac{d^{2}V_{2}}{d{\bar{y}}^{2}}-sV_{2}=0\;\mathrm{for}-1\leq y\leq 0$$

(A4) $${V_{1}|}_{y=0}={V_{2}|}_{y=0},\;{\frac{d^{}V_{1}}{dy}|}_{y=0}={\frac{d^{}V_{2}}{dy}|}_{y=0},\;{V_{2}|}_{y=-1}=0,\;{V_{1}|}_{y=1}=0$$

The solution to the above ODEs (A2)–(A3) takes the following forms:

(A5) $$V_{1}=C\cosh(Ky)+E\cosh(\sqrt{s}y)+F\sinh(\sqrt{s}y)\;\mathrm{for}\;0\leq y\leq 1$$

(A6) $$V_{2}=M\cosh(\sqrt{s}y)+F\sinh(\sqrt{s}y)\;\mathrm{for}-1\leq y\leq 0$$

Combining the boundary Equation (A4),

(A7) $$C=\frac{GE_{z}\zeta }{s(K^{2}-s)\cosh(K)},E=\frac{-GE_{z}\zeta }{2s(K^{2}-s)\cosh\sqrt{s}}\left(1+\frac{\cosh\sqrt{s}}{\cosh(K)}\right)\;F=\frac{GE_{z}\zeta }{2s(K^{2}-s)\sinh\sqrt{s}}\left(\frac{\cosh\sqrt{s}}{\cosh(K)}-1\right),M=\frac{GE_{z}\zeta }{s(K^{2}-s)\cosh(K)}+E$$

Applying the Laplace inverse transform to Equations (A5) and (A6) yields

(A8) $$v_{i}^{N}={\text{ℒ}}^{-1}\left[V_{i}\right]=\frac{1}{2\pi I}\int_{\sigma -I\infty }^{\sigma +I\infty }V_{i}e^{st}ds$$

in which *I* is the imaginary unit, *σ* is a real number satisfying *σ* > 0, and the integral path is presented in Figure A1. Based on the residue theorem, Equation (A8) equals

(A9) $$v_{1}^{N}=\sum_{k}\mathrm{Res}[V_{1}(s)e^{st},s_{k}],\;\mathrm{for}\;0\leq y\leq 1$$

(A10) $$v_{2}^{N}=\sum_{k}\mathrm{Res}[V_{2}(s)e^{st},s_{k}],\;\mathrm{for}-1\leq y\leq 0$$

where Res[*V_i_*(s)*e^st^, s_k_*] implies the residue of *V_i_*(*s*)*e^st^* at the isolated singularities, *s_k_*. The isolated singularities *s_k_* are enclosed in the simple closed curve *C_R_* + *L*, as shown in Figure A1. According to Equations (A5)–(A7), *s_k_* = 0, *K*^2^, –(2*P*–1)^2^π^2^/4, and –(*Pπ*)^2^, with *k* = 1, 2, 3, 4 and *P* = 1, 2, …. Consequently, the residues of *V_i_*(*s*)*e^st^* are evaluated as follows:

(A11) $$\mathrm{Res}[V_{1}(s)e^{st},0]=\underset{s_{1}\to 0}{\lim}\frac{d[s^{2}\cdot V_{1}(s)\cdot e^{st}]}{ds}=\frac{GE_{z}\zeta }{K^{2}}\left[\frac{1-\cosh(K)}{2\cosh(K)}(y-1)+\frac{\cosh(Ky)}{\cosh(K)}-1\right]$$

(A12) $$\mathrm{Res}[V_{2}(s)e^{st},0]=\underset{s_{1}\to 0}{\lim}\frac{d[s^{2}\cdot V_{2}(s)\cdot e^{st}]}{ds}=\frac{GE_{z}\zeta }{K^{2}}\left[\frac{1-\cosh(K)}{2\cosh(K)}(y-1)+\frac{1}{\cosh(K)}-1\right]$$

(A13) $$\mathrm{Res}[V_{i}(s)e^{st},K^{2}]=\underset{s_{2}\to K^{2}}{\lim}(s-K^{2})\cdot V_{i}(s)\cdot e^{st}=0$$

(A14) $$\mathrm{Res}\left[V_{i}(s)e^{st},-\frac{{\left[(2P-1)\pi \right]}^{2}}{4}\right]=\underset{s_{3P}\to -\frac{{\left[(2P-1)\pi \right]}^{2}}{4}}{\lim}\left[s+\frac{{\left[(2P-1)\pi \right]}^{2}}{4}\right]\cdot V_{i}(s)\cdot e^{st}\;=\frac{8GE_{z}\zeta {\left(-1\right)}^{P+1}e^{-{\left(2P-1\right)}^{2}\pi^{2}t/4}}{\left(2P-1)\pi [4K^{2}+{\left(2P-1\right)}^{2}\pi^{2}\right]}\cos\left[\frac{(2P-1)\pi y}{2}\right]$$

(A15) $$\mathrm{Res}\left[V_{i}(s)e^{st},-{\left(P\pi \right)}^{2}\right]=\underset{s_{4P}\to -{\left(P\pi \right)}^{2}}{\lim}\left[s+{\left(P\pi \right)}^{2}\right]\cdot V_{i}(s)\cdot e^{st}\;=\frac{GE_{z}\zeta [1/\cosh(K)-{\left(-1\right)}^{P+1}]e^{-{\left(P\pi \right)}^{2}t}}{P\pi [K^{2}+(P\pi^{2})]}\sin(P\pi y)$$

with *i* = 1, 2. According to Equations (A9) and (A10), summarizing Equations (A11)–(A15) produces the analytical velocities for two-layer Newtonian fluid EOF, which are exactly expressed by Equations (34) and (35).

In addition, the coefficients in Equations (38) and (39) are presented as follows:

$$\begin{array}{l} A_{1}=\frac{k_{f1}GE_{z}\zeta [1-\cosh(K)]}{12K^{2}\cosh(K)k_{eff1}}\cdot \frac{\left(2+S+Br\Gamma_{1}+m_{r}Br\Gamma_{2}\right)}{v_{ms1}+{\left(\rho c_{p}\right)}_{r}v_{ms2}},\\ A_{2}=\frac{k_{f1}GE_{z}\zeta }{K^{4}\cosh(K)k_{eff1}}\cdot \frac{\left(2+S+Br\Gamma_{1}+m_{r}Br\Gamma_{2}\right)}{v_{ms1}+{\left(\rho c_{p}\right)}_{r}v_{ms2}},\\ A_{3}=-\frac{k_{f1}}{2k_{eff1}}\left[\frac{GE_{z}\zeta [1+\cosh(K)]}{2K^{2}\cosh(K)}\cdot \frac{\left(2+S+Br\Gamma_{1}+m_{r}Br\Gamma_{2}\right)}{v_{ms1}+{\left(\rho c_{p}\right)}_{r}v_{ms2}}+S+\frac{Br{\left(GE_{z}\zeta \right)}^{2}{\left[1-\cosh(K)\right]}^{2}}{4K^{4}\cosh{\left(K\right)}^{2}}\right]\\ A_{4}=-\frac{k_{f1}Br{\left(GE_{z}\zeta \right)}^{2}[1-\cosh(K)]}{K^{5}\cosh{\left(K\right)}^{2}k_{eff1}},A_{5}=-\frac{k_{f1}Br{\left(GE_{z}\zeta \right)}^{2}}{4K^{2}\cosh{\left(K\right)}^{2}k_{eff1}},\\ B_{1}=\frac{k_{f1}GE_{z}\zeta [1-\cosh(K)]}{12K^{2}\cosh(K)k_{eff1}}\cdot \frac{\left(2+S+Br\Gamma_{1}+m_{r}Br\Gamma_{2}\right)}{v_{ms1}/{\left(\rho c_{p}\right)}_{r}+v_{ms2}},\\ B_{2}=\frac{k_{f1}}{2k_{eff1}}\left[\frac{GE_{z}\zeta [1-\cosh(K)]}{2K^{2}\cosh(K)}\cdot \frac{\left(2+S+Br\Gamma_{1}+m_{r}Br\Gamma_{2}\right)}{v_{ms1}+v_{ms2}{\left(\rho c_{p}\right)}_{r}}-m_{r}Br\Phi_{2}\right],\\ D_{1}=-\frac{k_{eff2}\left(d_{1}-d_{2}\right)}{k_{eff1}+k_{eff2}},\\ D_{2}=-\frac{k_{eff2}}{k_{eff1}+k_{eff2}}\left(\frac{k_{eff1}}{k_{eff2}}d_{1}+d_{2}\right),\\ D_{3}=\frac{k_{eff1}}{k_{eff2}}(KA_{4}+D_{1}),\\ D_{4}=(A_{2}+A_{5}/K^{2}+D_{2}),\\ d_{1}=A_{1}+A_{2}\cosh(K)+A_{3}+A_{4}\sinh(K)+A_{5}\left[\frac{\cosh{\left(K\right)}^{2}}{K^{2}}-1\right]\\ d_{2}=\left(-B_{1}+B_{2}-\frac{k_{eff1}}{k_{eff2}}KA_{4}+A_{2}+\frac{A_{5}}{K^{2}}\right). \end{array}$$
